# ROS-induced cleavage of NHLRC2 by caspase-8 leads to apoptotic cell death in the HCT116 human colon cancer cell line

**DOI:** 10.1038/s41419-017-0006-7

**Published:** 2017-12-14

**Authors:** Kensuke Nishi, Yuri Iwaihara, Toshiyuki Tsunoda, Keiko Doi, Toshifumi Sakata, Senji Shirasawa, Shuhei Ishikura

**Affiliations:** 1 Department of Cell Biology, Faculty of Medicine, Fukuoka University, Fukuoka, 814-0180 Japan; 2 Department of Otorhinolaryngology, Faculty of Medicine, Fukuoka University, Fukuoka, 814-0180 Japan; 3 Center for Advanced Molecular Medicine, Fukuoka University, Fukuoka, 814-0180 Japan

## Abstract

Excess production of reactive oxygen species (ROS) is known to cause apoptotic cell death. However, the molecular mechanisms whereby ROS induce apoptosis remain elusive. Here we show that the NHL-repeat-containing protein 2 (NHLRC2) thioredoxin-like domain protein is cleaved by caspase-8 in ROS-induced apoptosis in the HCT116 human colon cancer cell line. Treatment of HCT116 cells with the oxidant *tert*-butyl hydroperoxide (tBHP) induced apoptosis and reduced NHLRC2 protein levels, whereas pretreatment with the antioxidant *N*-acetyl-l-cysteine prevented apoptosis and the decrease in NHLRC2 protein levels seen in tBHP-treated cells. Furthermore, the ROS-induced decrease in NHLRC2 protein levels was relieved by the caspase inhibitor z-VAD-fmk. We found that the thioredoxin-like domain of NHLRC2 interacted with a proenzyme form of caspase-8, and that caspase-8 cleaved NHLRC2 protein at Asp580 *in vitro*. Furthermore, siRNA-mediated knockdown of caspase-8 blocked the ROS-induced decrease in NHLRC2 protein levels. Both shRNA and CRISPR-Cas9-mediated loss of NHLRC2 resulted in an increased susceptibility of HCT116 cells to ROS-induced apoptosis. These results suggest that excess ROS production causes a caspase-8-mediated decrease in NHLRC2 protein levels, leading to apoptotic cell death in colon cancer cells, and indicate an important role of NHLRC2 in the regulation of ROS-induced apoptosis.

## Introduction

Reactive oxygen species (ROS), such as hydrogen peroxide and the hydroxyl radical, are generated from normal metabolic processes or toxic insults^1,2^. ROS at low levels can act as cell signaling molecules by reversibly oxidizing thiol groups in proteins, thereby modifying protein structure and function. On the other hand, high levels of ROS can harm cells by nonspecifically damaging macromolecules, including DNA, proteins, and lipids. Therefore, maintenance of ROS homeostasis is essential for the regulation of a variety of cellular processes, such as cell survival, death, and proliferation^1,2^.

ROS are implicated in the induction or enhancement of apoptosis^3,4^. Regulation and execution of apoptosis are mainly accomplished by caspases, a family of aspartic acid-specific cysteine proteases^5,6^. Caspase-mediated cleavage of a large set of specific proteins is responsible for most apoptotic changes^7^. On the basis of structural and functional characteristics, caspases involved in apoptosis are grouped into initiator (caspase-2, -8, -9, and -10) and effector (caspase-3, -6, and -7) caspases^8^. Apoptosis is initiated by extracellular and intracellular signals via two main pathways, the death receptor-mediated (extrinsic) and the mitochondria-mediated (intrinsic) pathways^8^. Among caspase family members, caspase-8 is a key mediator of apoptotic signals associated with death receptors^9–11^, whereas caspase-9 has a pivotal role in mitochondria-mediated apoptosis^12^. Both caspase-8 and -9 are involved in ROS-induced apoptosis^13–16^, although ROS production is thought to induce apoptosis mainly through the intrinsic pathway.

Under normal physiological conditions, intracellular levels of ROS are strictly maintained to prevent cell damage^2^. Detoxification of ROS is facilitated by non-enzymatic molecules or through antioxidant enzymes. Thioredoxin (Trx), which is present in every organism, is one of the important antioxidant proteins^17,18^. The catalytic site of Trx contains a Cys–X–X–Cys motif, where X represents any amino acid^19,20^. These two cysteines are cycled between an active (reduced) dithiol form and an inactive (oxidized) disulfide form. In its active state, Trx scavenges ROS and keeps proteins in their reduced state. Trx, which constitutes a key component of a redox regulatory system in mammalian cells, is the best characterized regulator of the thiol group in proteins^21^. However, there are numerous other Trx-like proteins, whose functions and interactors are unknown^22^.

NHL-repeat-containing protein 2 (NHLRC2) is a 79 kDa protein of 726 amino acids that consists of the N-terminal Trx-like domain and a C-terminal NHL (NCL-1, HT2A, and LIN-41)-repeat domain. The NHL-repeat domains have been proposed to form β-propeller structures^23,24^, similar to those of the WD40-repeat domain, suggesting their functions in protein–protein interactions. However, interactors of NHLRC2 have not been identified yet. The *NHLRC2* gene has been found in many species, from flies to humans, and their sequences are highly conserved during evolution. A recent study reported that a mutation in the *NHLRC2* gene in cattle was related to embryonic malformation^25^, suggesting an important role in embryonic development. Based on the amino acid sequence, NHLRC2 is expected to be involved in the regulation of redox states of particular proteins. However, the roles and interactors of NHLRC2 are as yet unexplored.

In this study, we report that NHLRC2 is cleaved by caspase-8 in ROS-induced apoptosis in the HCT116 human colon cancer cell line. We found that NHLRC2 protein levels were decreased in ROS-induced apoptosis in HCT116 cells. Caspase-8 was identified as the enzyme responsible for the decreased NHLRC2 levels in ROS-induced apoptosis. Furthermore, we show that loss of NHLRC2 resulted in an increased susceptibility of HCT116 cells to ROS-induced apoptosis. Taken together, these results suggest that excess ROS production causes a caspase-8-mediated decrease in NHLRC2 protein levels, leading to apoptotic cell death in colon cancer cells, indicating an important role for NHLRC2 in the regulation of ROS-induced apoptosis.

## Results

### The oxidant tBHP reduces NHLRC2 protein levels through ROS production in HCT116 cells

**Fig. 1:**
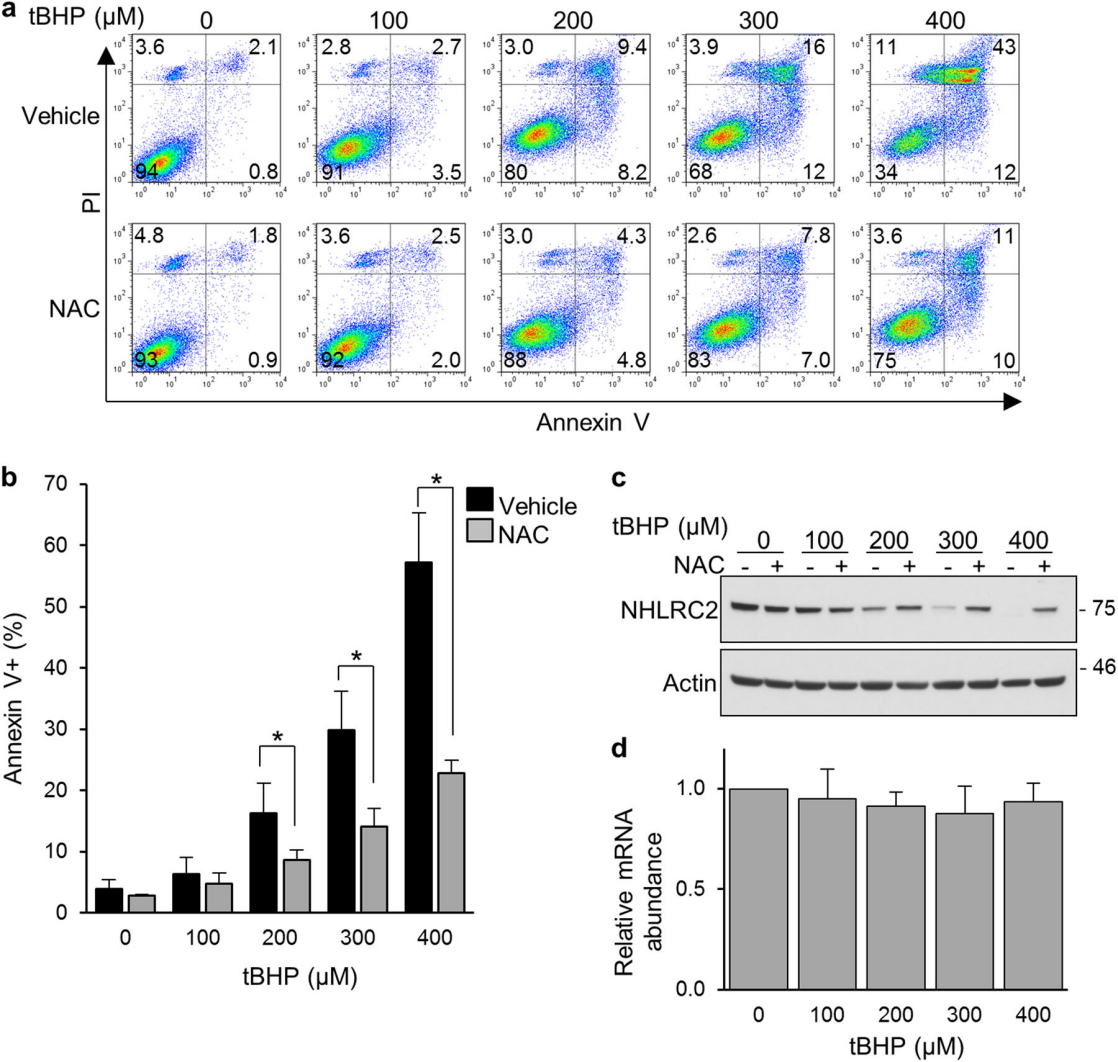
The oxidant tBHP reduces NHLRC2 protein levels through ROS production in HCT116 cells **a**, **b** Percentages of cells that underwent apoptosis for HCT116 cells treated with tBHP and NAC. **a** Numbers adjacent to the outline indicate the percentage of cells in each area. **b** The sum of annexin V^+^PI^−^ and annexin V^+^PI^+^ populations in **a** is represented as the percentage of annexin V^+^ cells. Data represent the mean ± SD based on three independent experiments. *P < 0.05. **c** Immunoblotting analysis of NHLRC2 in HCT116 cells treated with tBHP and NAC. Actin was used as a loading control. **d** Quantitative RT-PCR analysis of *NHLRC2* gene in HCT116 cells treated with tBHP. The mRNA expression levels of *NHLRC2* were normalized against those of *β-actin*. The results are shown as the values relative to 0 µM tBHP cells. **a**–**d** Data are representative of three independent experiments

To study the potential role of NHLRC2 in ROS-induced apoptosis, we examined the effects of the oxidant *tert*-butyl hydroperoxide (tBHP) on apoptotic cell death and NHLRC2 protein levels in HCT116 cells. The HCT116 human colon cancer cell line is a widely used cell line that is sensitive to a number of apoptosis-inducing agents^26–29^. Here we used tBHP to induce ROS production in the cells, because it is widely used as a better alternative to H_2_O_2_ in oxidative stress studies^30–32^. Treatment of HCT116 cells with tBHP caused a dose-dependent increase in the population of annexin V-positive apoptotic cells (Fig. 1a, b). On the other hand, pretreatment with the antioxidant *N*-acetyl-l-cysteine (NAC) blocked the tBHP-induced increase in apoptotic cells (Fig. 1a, b). Furthermore, treatment with tBHP resulted in a marked decrease in NHLRC2 protein levels, whereas pretreatment with NAC relieved the tBHP-induced decrease in NHLRC2 protein levels (Fig. 1c). The mRNA levels of *NHLRC2* gene were not affected by tBHP treatment (Fig. 1d). These results indicated that tBHP treatment induced apoptotic cell death and reduced NHLRC2 protein levels through ROS production in HCT116 cells.

### Caspases are involved in the ROS-induced decrease in NHLRC2 protein levels

**Fig. 2:**
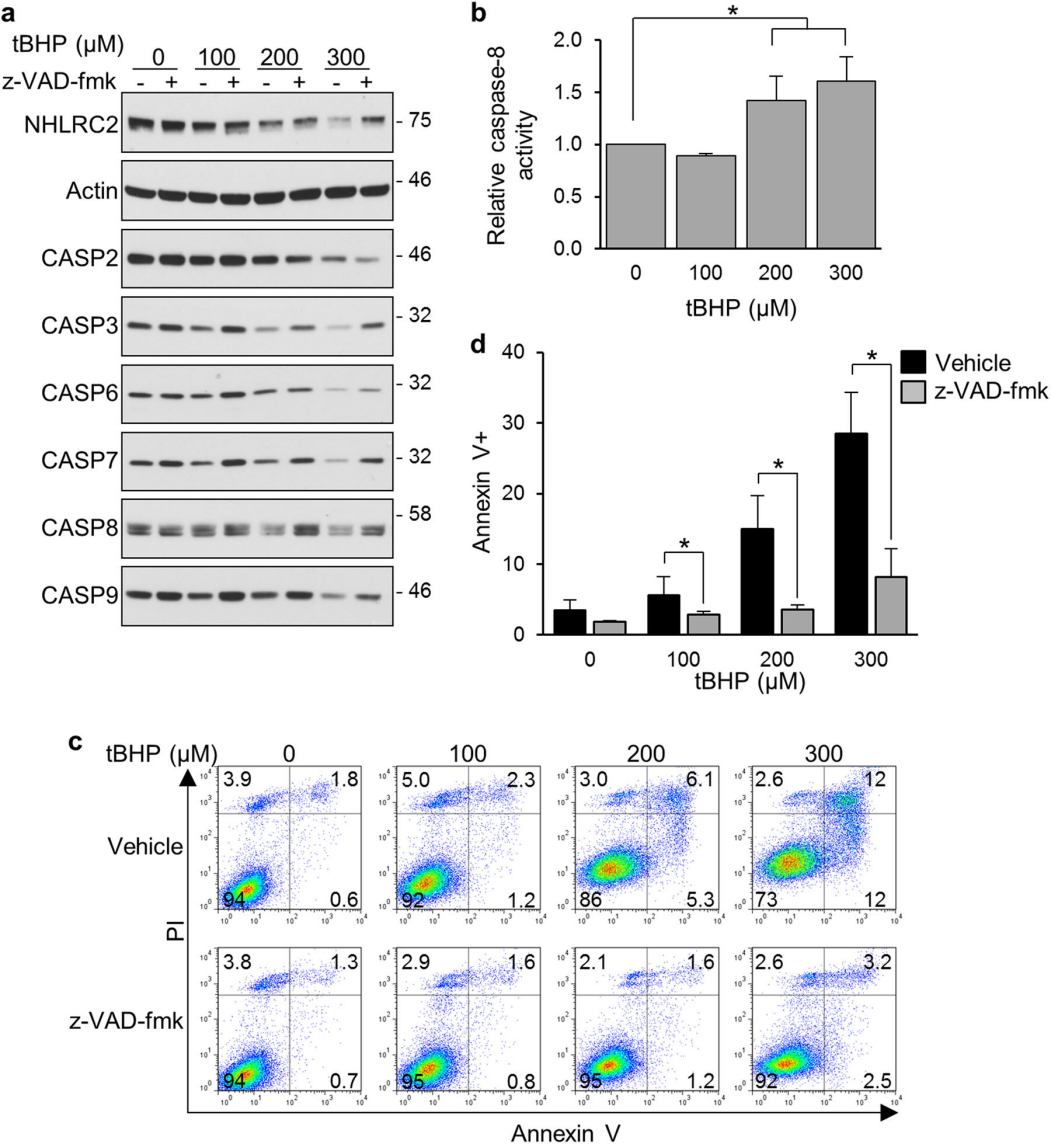
Inhibition of caspase activity prevents ROS-induced decrease in NHLRC2 protein levels in HCT116 cells **a** Immunoblotting analysis of NHLRC2 and caspases in HCT116 cells treated with tBHP and z-VAD-fmk. **b** Caspase-8 activity in HCT116 cells treated with tBHP. The results are shown as the values relative to 0 µM tBHP cells. **c**, **d** Percentages of cells that underwent apoptosis for HCT116 cells treated with tBHP and z-VAD-fmk. **c** Numbers adjacent to the outline indicate the percentage of cells in each area. **d** The sum of annexin V^+^PI^−^ and annexin V^+^PI^+^ populations in **c** is represented as the percentage of annexin V^+^ cells. Data represent the mean ± SD based on three independent experiments. *P < 0.05. **a**–**d** Data are representative of three independent experiments

Caspases have a critical role in protein cleavage in the apoptotic pathway. To find out whether caspases were involved in the decreased NHLRC2 protein levels in ROS-induced apoptosis, we first examined the activation of caspases in tBHP-treated HCT116 cells. We assessed caspase activation by the disappearance of proenzyme using immunoblotting analysis. It was difficult to detect the large and small subunits of processed caspases under the conditions of these assays, probably due to the short half-life of the active caspases in the cells. Treatment of HCT116 cells with tBHP caused a dose-dependent decrease in the proenzyme levels of initiator caspases (caspase-2, -8, and -9) and effector caspases (caspase-3, -6, and -7) (Fig. 2a). Furthermore, activation of caspase-8 by tBHP treatment was confirmed by measuring caspase-8 activity in HCT116 cells (Fig. 2b). These results indicated that tBHP treatment induced activation of both caspases that are involved in the intrinsic or extrinsic pathway. We next tested the effects of z-VAD-fmk, a caspase inhibitor with broad specificity, on apoptosis and the decreased NHLRC2 protein levels in tBHP-treated HCT116 cells. The z-VAD-fmk relieved the tBHP-induced decrease in NHLRC2 protein levels with concomitant restoration of procaspase levels, except for procaspase-2 levels (Fig. 2a). Furthermore, tBHP-induced apoptosis was efficiently abrogated by pretreatment with z-VAD-fmk (Fig. 1c, d). These results suggested an involvement of caspases in the decreased NHLRC2 protein levels in ROS-induced apoptosis.

### Overexpression of caspase-1, -2, -4, -8, -9, or -10 dramatically decreases NHLRC2 protein levels

**Fig. 3:**
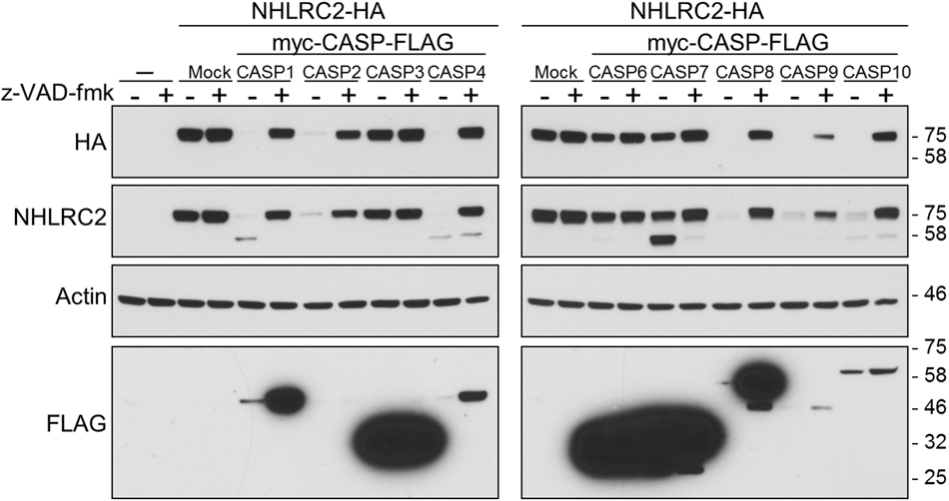
Overexpression of caspase-1, -2, -4, -8, -9, or -10 dramatically diminishes NHLRC2-HA protein Immunoblotting analysis of NHLRC2-HA and myc-CASP-FLAG proteins in 293FT cells. Actin was used as a loading control. Data are representative of three independent experiments

To examine whether caspases cleave NHLRC2 protein in ROS-induced apoptosis, NHLRC2 tagged with an HA-tag at the C terminus (NHLRC2-HA) was co-overexpressed individually with a series of caspases tagged with myc and FLAG tags (myc-caspase-FLAG) at the N and C termini, respectively, in 293FT cells. Expression levels of these proteins were examined by immunoblotting analysis. Overexpression of procaspases is sufficient to induce their autoactivation^33–35^. As shown in Fig. 3, expression of myc-caspase-FLAG proteins for caspase-1, -2, -4, -8, -9, and -10 was hardly observed in the absence of z-VAD-fmk, whereas the treatment with z-VAD-fmk yielded an increase in these caspases’ proenzyme levels, indicating that these myc-caspase-FLAG proteins were expressed as active forms (Fig. 3). Immunoblotting analysis using an anti-HA antibody showed that co-overexpression with caspase-1, -2, -4, -8, -9, or -10, but not caspase-3, -6, or -7, resulted in a marked decrease in NHLRC2-HA protein levels (Fig. 3). Furthermore, treatment with z-VAD-fmk blocked the decrease in NHLRC2-HA protein levels by co-overexpression of these caspases (Fig. 3), indicating that enzyme activity of caspases was required to achieve the dramatic decrease in NHLRC2 protein levels. It was reported that activated caspases cleaved a DVPD sequence within an HA-tag, causing loss of immunoreactivity against the anti-HA antibody^36^. Therefore, NHLRC2-HA protein levels were also assessed by immunoblotting using anti-NHLRC2 polyclonal antibody raised against full-length human NHLRC2 protein. Immunoblotting analysis using an anti-NHLRC2 antibody showed similar results to those with the anti-HA antibody (Fig. 3), indicating that co-overexpression of caspase-1, -2, -4, -8, -9, or -10 caused the marked decrease in NHLRC2-HA protein levels, but not the cleavage of the HA-tag. Additional bands corresponding to NHLRC2 fragments cleaved by caspase-1, -2, -4, -8, -9, or -10 were barely detected in immunoblotting analysis, suggesting that the cleaved forms of NHLRC2 by these caspases were rapidly degraded in the cells. On the other hand, in the immunoblotting analysis using an anti-NHLRC2 antibody, co-overexpression of caspase-7 resulted in the appearance of an additional 58 kDa band with a concomitant slight decrease in a 78 kDa band corresponding to full-length NHLRC2, indicating that the cleavage by caspase-7 produced a stable cleaved form of NHLRC2. These results suggested that cleavage sites of NHLRC2 may be different between caspase-1, -2, -4, -8, -9, or -10 and caspase-7, resulting in cleaved forms with different stabilities.

### The Trx-like domain of NHLRC2 interacts with proenzyme forms of caspase-1, -2, -4, -8, -9, and -10

**Fig. 4:**
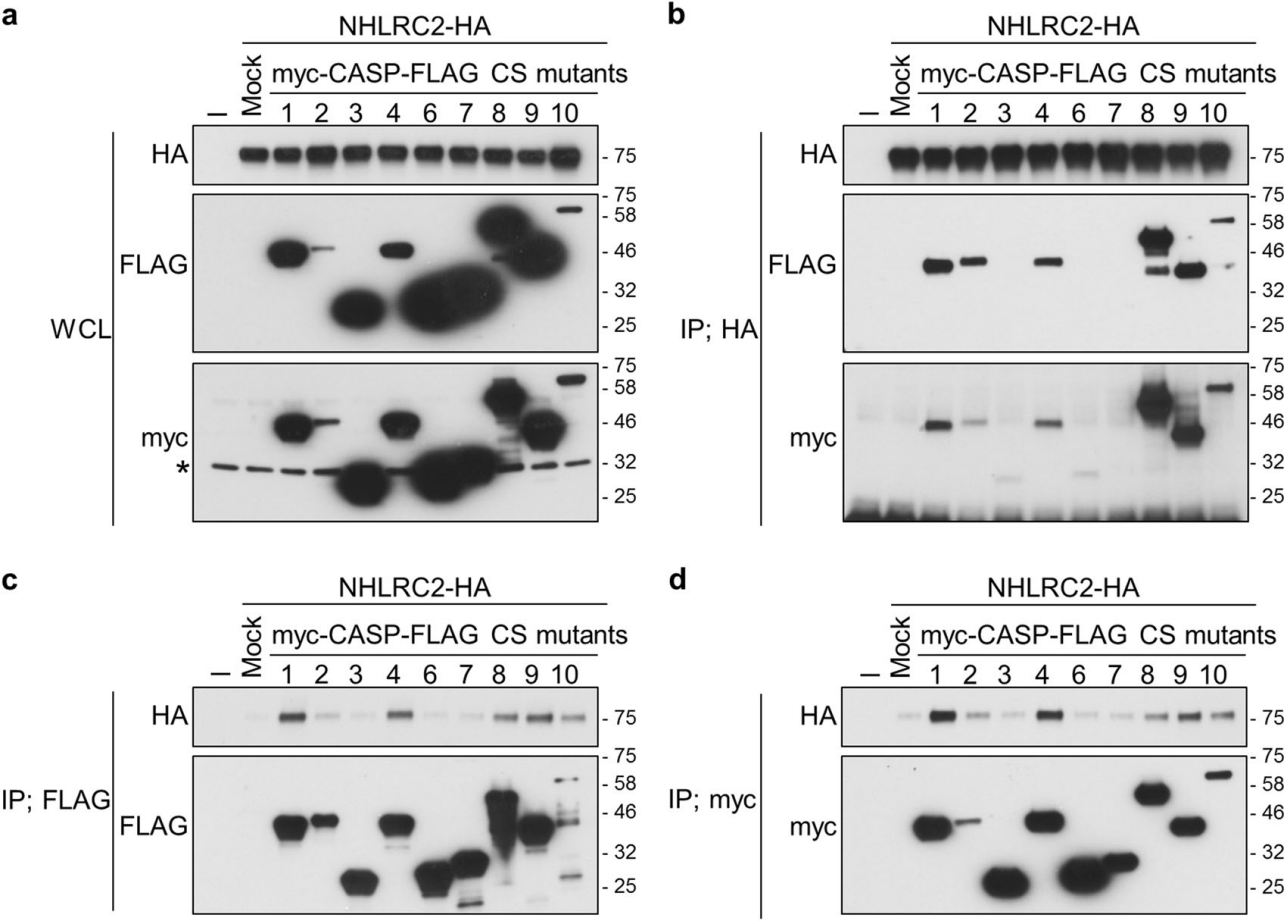
NHLRC2 interacts with the catalytically inactive forms of caspase-1, -2, -4, -8, -9, and -10 **a**–**d** Co-immunoprecipitation analysis of NHLRC2-HA and myc-CASP-FLAG CS mutants. Data are representative of three independent experiments. * Indicates endogenous c-myc protein. IP, immunoprecipitation; WCL, whole-cell lysate

To clarify whether caspases recognize NHLRC2 as a substrate, we investigated the interaction between NHLRC2 and caspases. As co-overexpression with caspases resulted in the dramatic decrease in NHLRC2-HA protein levels described above, we substituted the catalytic cysteine residue in each myc-caspase-FLAG with serine to express catalytically inactive forms of caspases (myc-caspase-FLAG CS mutants). The CS mutant of each caspase was co-overexpressed with NHLRC2-HA in 293FT cells. Expression of myc-caspase-FLAG CS mutants was easily detected by both anti-FLAG and anti-myc antibodies, unlike that of their wild-type proteins, indicating that the CS mutants were expressed as catalytically inactive forms (Fig. 4a). Nonetheless, the intensity of bands corresponding to caspase-2 and -10 CS mutants was remarkably lower compared with those of other caspases, due to a low solubility in Co-IP buffer, by the formation of large aggregates in the cells (see below). The CS mutants of caspase-1, -2, -4, -8, -9, and -10 were co-immunoprecipitated together with NHLRC2-HA by anti-HA antibody (Fig. 4b), whereas co-immunoprecipitation with the CS mutants of caspase-3, -6, and -7 was not observed. Furthermore, reciprocal co-immunoprecipitation assays using anti-FLAG or anti-myc antibody also revealed that NHLRC2-HA interacted specifically with the CS mutants of caspase-1, -2, -4, -8, -9, and -10 (Fig. 4c, d).

**Fig. 5:**
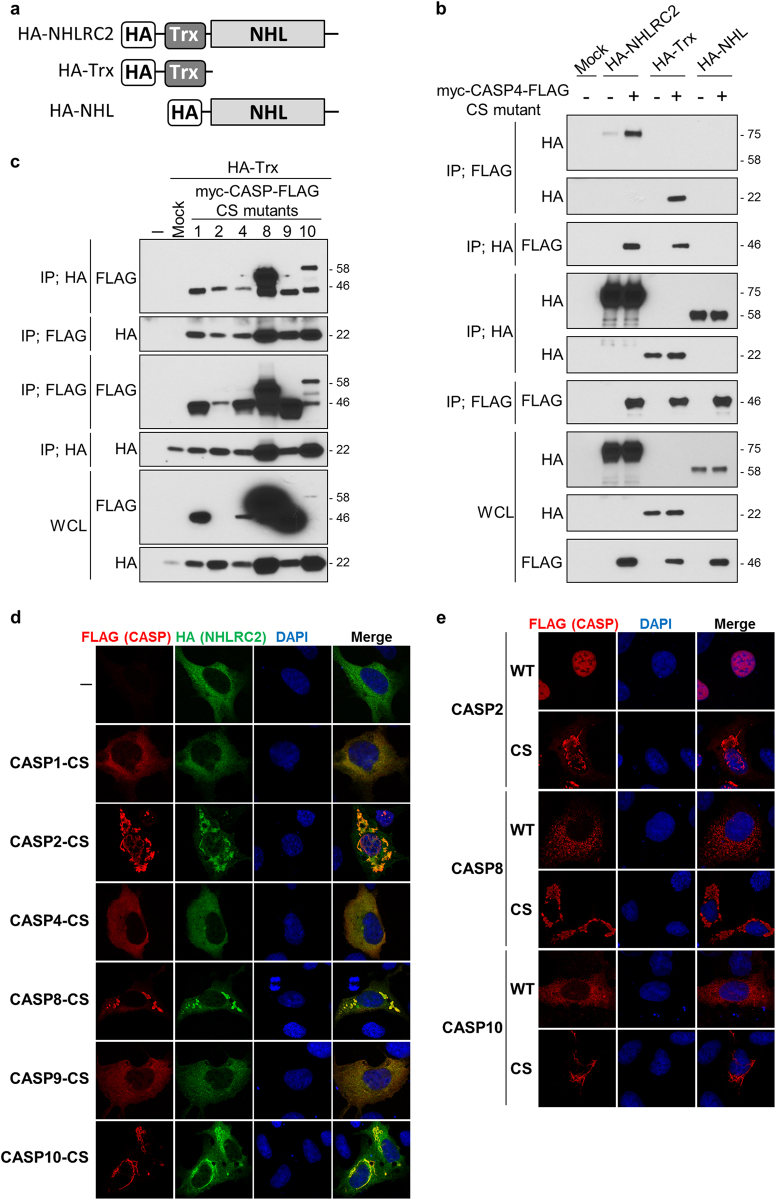
The Trx-like domain of NHLRC2 interacts with catalytically inactive caspases **a** Schematic illustration of HA-NHLRC2, HA-Trx, and HA-NHL. HA, HA-tag; NHL, NHL-repeat domain; Trx, thioredoxin-like domain. **b** Co-immunoprecipitation analysis of HA-NHLRC2, HA-Trx, or HA-NHL with myc-CASP4-FLAG CS mutant. **c** Co-immunoprecipitation analysis of HA-Trx and myc-CASP-FLAG CS mutants. **d**, **e** Immunofluorescence images for NHLRC2-HA and wild-type and catalytically inactive (CS) myc-CASP-FLAG in HEK293 cells. **b**–**e** Data are representative of three independent experiments

To identify the region of NHLRC2 that is required for interaction with caspases, we tested truncated forms of NHLRC2 protein for their ability to bind to myc-caspase-4-FLAG CS mutant (Fig. 5a). Myc-caspase-4-FLAG CS mutant interacted specifically with the Trx-like domain, but not the NHL-repeat domain, of NHLRC2 (Fig. 5b). Furthermore, interactions between the Trx-like domain of NHLRC2 and the CS mutants of caspase-1, -2, -8, -9, and -10 were also observed (Fig. 5c). These results suggested that the Trx-like domain of NHLRC2 bound to the proenzyme forms of caspase-1, -2, -4, -8, -9, and -10.

To further confirm the interaction between NHLRC2 and caspases-1, -2, -4, -8, -9, and -10, we examined the localization of NHLRC2-HA and myc-caspase-FLAG CS mutants in HEK293 cells by immunofluorescence analysis. NHLRC2-HA was distributed in the cytosol when overexpressed alone in the cells (Fig. 5d, top panels). CS mutants of caspase-2, -8, and -10 formed large aggregates in the cytosol, whereas wild-type caspase-2, and caspase-8 and -10 were localized in the nucleus and cytosol, respectively (Fig. 5e). Interestingly, NHLRC2-HA that was co-overexpressed with the CS mutant of caspase-2, -8 or -10 was incorporated into their aggregates (Fig. 5d). On the other hand, co-overexpression with CS mutants of caspase-1, -4, or -9 did not affect the cytosolic distribution of NHLRC2-HA, and these CS mutants were distributed in the cytosol, similar to NHLRC2-HA (Fig. 5d). Together, these results suggested that the Trx-like domain of NHLRC2 interacted with proenzyme forms of caspase-1, -2, -4, -8, -9, and -10.

### Caspase-8 cleaves NHLRC2 protein at Asp580 in ROS-induced apoptosis

**Fig. 6:**
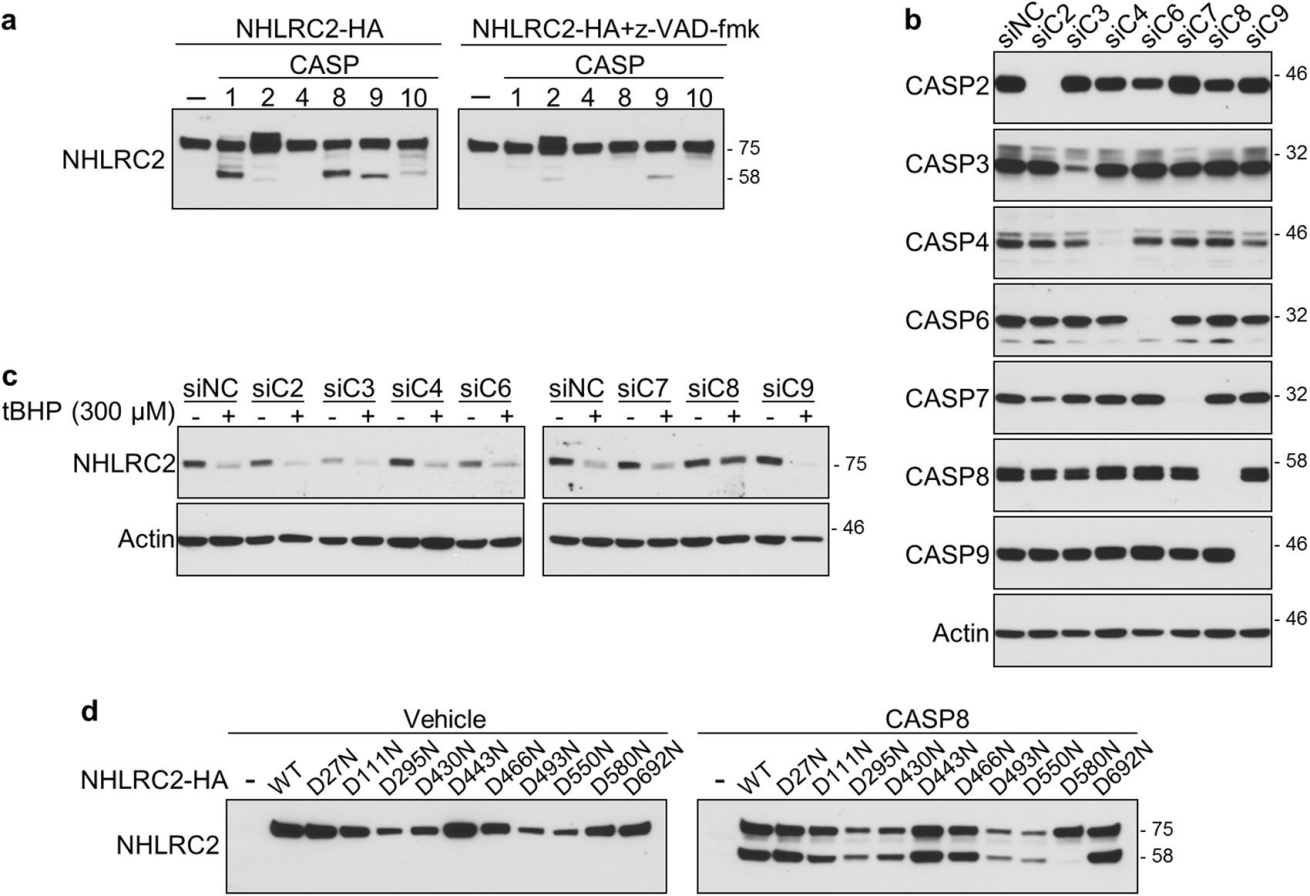
Caspase-8 cleaves NHLRC2 protein at Asp580 in ROS-induced apoptosis **a**, **d** Cell-free caspase cleavage assay of NHLRC2. **b**, **c** Immunoblotting analyses of caspases and NHLRC2 in HCT116 cells transfected with siRNAs for caspases. **a**–**d** Data are representative of three independent experiments

To identify the caspase that cleaves NHLRC2 protein, we carried out a cell-free caspase assay using active forms of recombinant caspase-1, -2, -4, -8, -9, and -10. Whole-cell lysates from 293FT cells transfected with expression vectors for NHLRC2-HA were incubated individually with each recombinant caspase. Cleavage of NHRLC2-HA was evaluated by immunoblotting using an anti-NHLRC2 antibody. As shown in Fig. 6a, additional bands of about 58 kDa were detected after incubation with caspase-1, -8, or -9, in addition to the main 78 kDa band corresponding to full-length NHLRC2-HA. The appearance of the 58 kDa band was suppressed significantly in the presence of z-VAD-fmk. These results indicated that caspase-1, -8, and -9 cleaved NHLRC2 in vitro. Interestingly, the mobilities of the bands that appeared after incubation with caspase-1, -8, or -9 were slightly different (Fig. 6a), suggesting that caspase-1, -8, and -9 cleaved NHLRC2 at different sites.

To identify the caspase responsible for the cleavage of NHRLC2 in ROS-induced apoptosis, we depleted a series of caspases in HCT116 cells using siRNA. We did not test siRNAs for caspase-1 and -10, because their expression was not observed in HCT116 cells by immunoblotting analysis (data not shown). Efficient knockdown of endogenous caspase proteins was confirmed by immunoblotting (Fig. 6b). Only knockdown of caspase-8 obviously prevented the ROS-induced decrease in NHLRC2 protein levels, whereas the depletion of other caspases did not restore the decreased NHRLC2 protein levels (Fig. 6c). These results indicated that caspase-8 cleaved NHLRC2 protein in ROS-induced apoptosis in HCT116 cells.

Caspases typically cleave at the C-terminal side of the last Asp residue in the tetra-peptide sequence X–X–X–D of the target protein. To identify the caspase-8 cleavage site, we introduced Asp to Asn mutations at several predicted candidate sites in the NHLRC2 sequence. These mutants were expressed in 293FT cells and subjected to the cell-free caspase assay using the active form of recombinant caspase-8. As shown in Fig. 6d, of the mutations introduced, only the mutation at Asp580 efficiently prevented the cleavage of NHLRC2 protein by caspase-8, indicating that caspase-8 cleaved NHLRC2 protein at Asp580.

### Loss of NHLRC2 increases the susceptibility of cells to apoptosis induced by ROS or etoposide

**Fig. 7:**
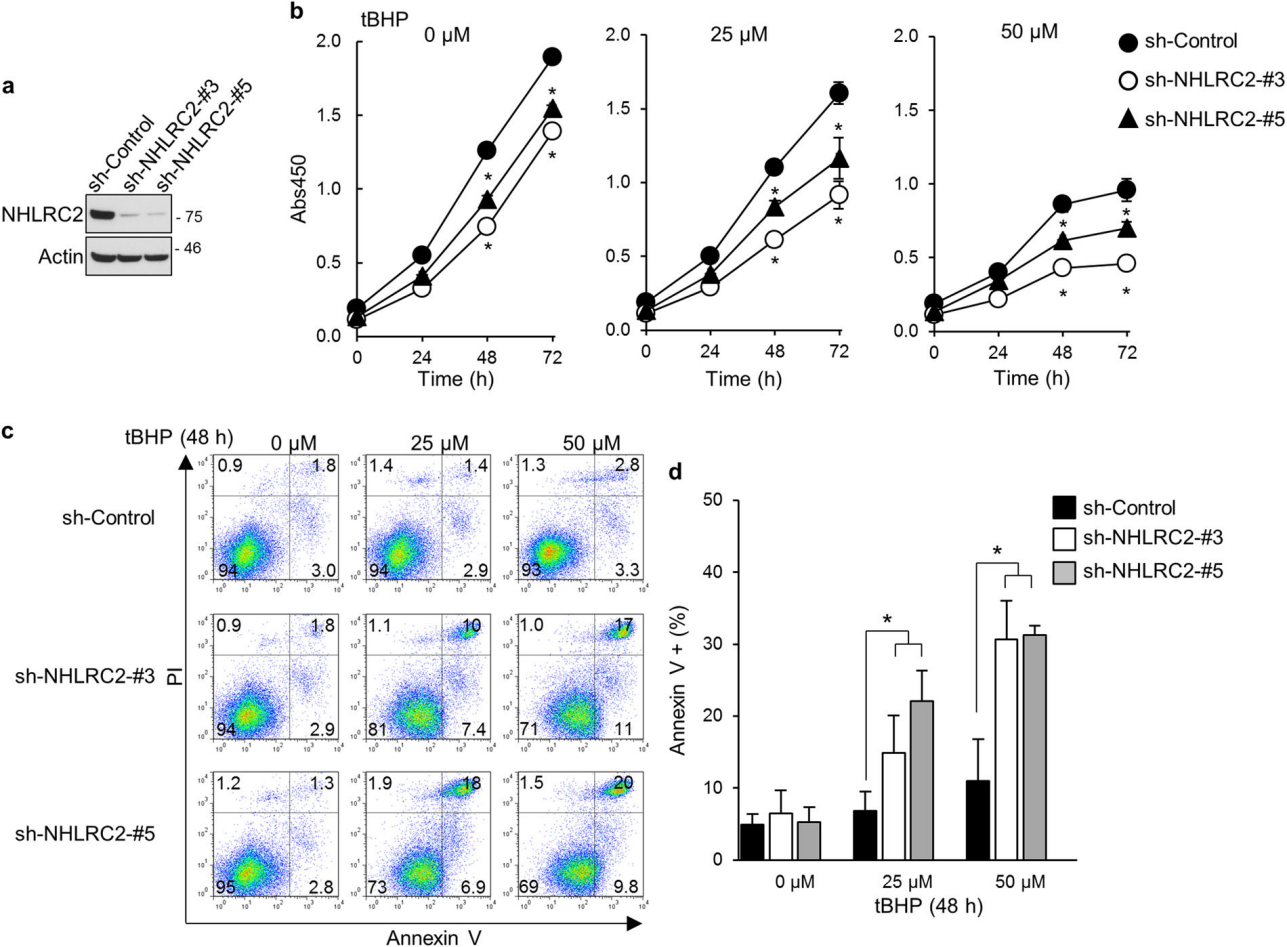
shRNA-mediated knockdown of NHLRC2 increases the susceptibility of HCT116 cells to ROS-induced apoptosis **a** Immunoblotting analysis of NHLRC2 in HCT116 cells transduced with retrovirus for shRNAs for control (sh-Control) or NHLRC2 (sh-NHLRC2-3 and -5). **b**–**d** Analyses of cell proliferation **b** and apoptosis **c**, **d** in HCT116 cells transduced with retrovirus for sh-Control, sh-NHLRC2-3 or -5. **b** *P < 0.05. **c** Numbers adjacent to the outline indicate the percentage of cells in each area. **d** The sum of annexin V^+^PI^−^ and annexin V^+^PI^+^ populations in **c** is represented as the percentage of annexin V^+^ cells. Data represent the mean ± SD based on three independent experiments. *P < 0.05. **a**–**d** Data are representative of three independent experiments

To clarify the significance of caspase-8-mediated cleavage of NHLRC2 protein in ROS-induced apoptosis, we knocked down NHLRC2 in HCT116 cells using retrovirus-mediated shRNA and examined the effects on cell proliferation and ROS-induced apoptosis. Endogenous NHLRC2 protein was knocked down efficiently by two different shRNAs (Fig. 7a). The decrease in NHLRC2 protein levels resulted in the suppression of cell proliferation regardless of the presence of tBHP (Fig. 7b). Furthermore, NHLRC2 knockdown did not induce apoptosis in the absence of tBHP but promoted apoptosis caused by tBHP at relatively low concentrations (Fig. 7c, d). To further confirm these results, a lentivirus-mediated CRISPR-Cas9 strategy was utilized. Endogenous NHLRC2 protein was efficiently depleted by two different sgRNAs (Supplementary Fig. 1a). In accordance with the results of shRNA-mediated knockdown, CRISPR-Cas9-mediated loss of NHLRC2 resulted in the suppression of cell proliferation and the increased susceptibility to apoptosis caused by tBHP at relatively low concentrations (Supplementary Fig. 1b-d). Together, these results suggested that the caspase-8-mediated decrease in NHLRC2 protein levels is an important step in ROS-induced apoptosis in HCT116 cells.

Furthermore, we examined an involvement of NHLRC2 in ROS-induced apoptosis in the HEK293 human embryonic kidney cells. Treatment of HEK293 cells with tBHP resulted in a marked decrease in NHLRC2 protein levels, whereas pretreatment with z-VAD-fmk relieved the tBHP-induced decrease in NHLRC2 protein levels (Supplementary Fig. 2a). Furthermore, shRNA-mediated knockdown of NHLRC2 increased the sensitivity of HEK293 cells to tBHP-induced apoptosis (Supplementary Fig. 2b-d). These results suggested that caspase-mediated downregulation of NHLRC2 is an important step in ROS-induced apoptosis in various cell types.

**Fig. 8:**
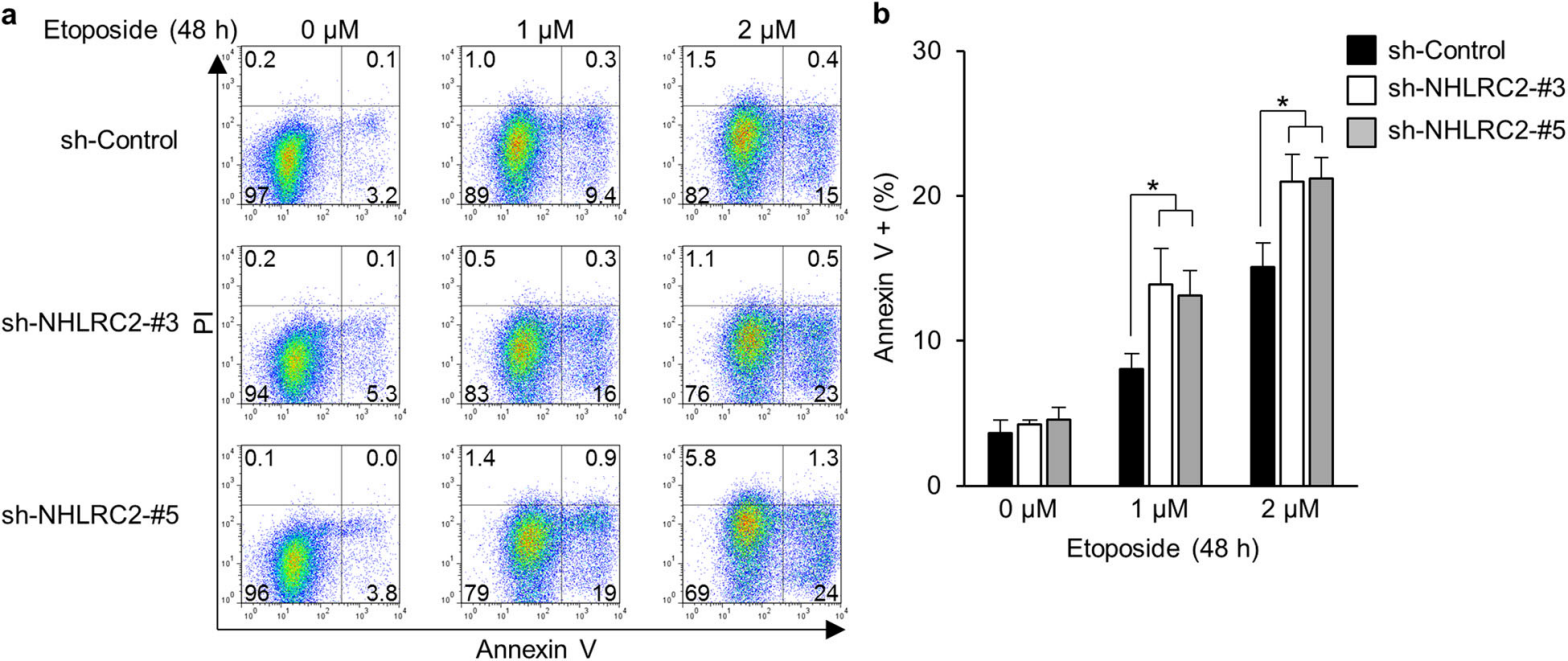
shRNA-mediated knockdown of NHLRC2 increases the susceptibility of HCT116 cells to etoposide-induced apoptosis **a**, **b** Analyses of apoptosis in HCT116 cells transduced with retrovirus for sh-Control, sh-NHLRC2-3, or -5. **a** Numbers adjacent to the outline indicate the percentage of cells in each area. **b** The sum of annexin V^+^PI^−^ and annexin V^+^PI^+^ populations in **a** is represented as the percentage of annexin V^+^ cells. Data represent the mean ± SD based on three independent experiments. *P < 0.05

NHLRC2 protein interacted with and was cleaved by not only caspase-8 but also caspase-1, -2, -4, -9, or -10 in vitro (Figs. 3–5), suggesting that NHLRC2 was involved in apoptosis induced by stimuli other than ROS. Therefore, we examined the effect of NHLRC2 loss on etoposide-induced apoptosis. Etoposide is known to cause DNA damage by inhibiting topoisomerase II, leading to apoptosis mainly through mitochondrial pathway^37,38^. shRNA-mediated knockdown of NHLRC2 promoted apoptosis induced by etoposide in HCT116 cells (Fig. 8a, b), suggesting that NHLRC2 may play an important role in various types of caspase-dependent cell death.

## Discussion

In this study, we demonstrated that the NHLRC2 Trx-like domain protein is a novel caspase-8 substrate that is involved in the regulation of ROS-induced apoptosis. We show that treatment with the oxidant tBHP caused a marked decrease in NHLRC2 protein levels in HCT116 cells. On the other hand, the tBHP-induced decrease in NHLRC2 protein levels was prevented by the ROS-scavenger NAC or the caspase inhibitor z-VAD-fmk, indicating that NHLRC2 protein levels are regulated through caspase activity in response to ROS production. We show that caspase-8 cleaved NHLRC2 in ROS-induced apoptosis. Furthermore, loss of NHLRC2 resulted in an increase in susceptibility of HCT116 cells to ROS-induced apoptosis. These results suggest that caspase-8-mediated cleavage of NHLRC2 protein is an important step in the ROS-induced apoptotic pathway in HCT116 cells, indicating a key role of NHLRC2 in ROS-induced apoptosis.

The cleavage of NHLRC2 protein by caspase-8 in vitro yielded an additional 58 kDa fragment detected by immunoblotting analysis (Fig. 6). In contrast, tBHP treatment of HCT116 cells resulted in a decrease in the levels of the 78 kDa band corresponding to full-length NHLRC2 without the appearance of additional bands (Figs. 1 and 2). Furthermore, co-overexpression of myc-caspase-8-FLAG dramatically decreased full-length NHLRC2-HA protein levels in 293FT cells; however, processed fragments of NHLRC2-HA were not observed (Fig. 3). Namely, fragment of NHLRC2 cleaved in in vitro experiment was detected, whereas fragment of NHLRC2 cleaved within the cells was not detected. These results suggest that cleaved fragments of NHLRC2 by caspase-8 may be further processed by other proteases within the cells.

Recently, Polkoff et al.^25^ reported that a mutation in a highly conserved protein-coding region of the *NHLRC2* gene in cattle is related to embryonic malformation. Furthermore, homozygous deletion of the *NHLRC2* gene in mice yielded an embryonic lethality^39^. On the other hand, NHLRC2 was identified as a blood biomarker for Alzheimer’s disease^40^. Therefore, it has been indicated that NHLRC2 plays an important role in embryonic development and is related to human diseases. However, the functions and physiological roles of NHLRC2 had been totally unexplored. In this study, we show that NHLRC2 acted as an antioxidant protein in the regulation of ROS-induced apoptosis. Furthermore, the depletion of NHLRC2 significantly suppressed cell proliferation in HCT116 cells, even in the absence of excessive ROS production. Thus, NHLRC2 may have an additional role in the regulation of cell proliferation, in addition to apoptosis.

Here we show that the Trx-like domain of NHLRC2 interacted with the proenzymes of caspases. In general, the two cysteine residues in the catalytic site of the Trx-like domain are thought to regulate redox states of thiol groups of proteins^19,20^. Caspases are a family of cysteine proteases that use a cysteine thiol group in the active site to cleave a peptide bond after an Asp residue of the target protein. Thus, NHLRC2 may participate in caspase activation by regulating the redox state of the catalytic cysteine thiol group of caspases.

NHL-repeat domains have been demonstrated to form β-propeller structures^23,24^ similar to those of the WD40-repeat domain, which is involved in protein–protein interactions. Many NHL-repeat domain proteins have additional motifs—including RING domains, B-box zinc finger domains and coiled-coil domains—indicating their diverse functions in various cellular pathways. For example, NHL-repeat-containing protein 1 (NHLRC1), which is a causative gene for Lafora disease, an autosomal recessive neurodegenerative disorder, encodes an E3 ubiquitin ligase that consists of a RING domain and an NHL-repeat domain^41,42^. The NHL-repeat domain of NHLRC1 has been shown to bind to the glucan phosphatase laforin. In contrast, the NHL-repeat domain of the *Drosophila* TRIM-NHL protein Brain tumor (Brat) has been reported to bind directly to *hunchback* RNA, leading to repression of its translation, suggesting a novel function of the NHL-repeat domain in translational regulation through RNA binding^43,44^. Among NHL-repeat domain proteins, NHLRC2 is the only known protein containing the Trx-like domain. However, interactors of the NHL-repeat domain of NHLRC2 have not yet been identified. To better understand the physiological role of NHLRC2, identifying proteins that interact with the NHL-repeat domain of NHLRC2 is important.

Maintenance of ROS homeostasis is recognized as a key process underlying the molecular basis of many diseases, such as cancers. In cancer cells, increased ROS generation, which results from elevated metabolic activity and mitochondrial dysfunction, contributes to tumor development and progression^1,4^. Cancer cells are more sensitive to rapid increases in ROS levels than normal cells. Indeed, ROS production is a common characteristic of many anticancer therapies^45^. In this study, we show that the downregulation of NHLRC2 expression caused an increased susceptibility of HCT116 cells to ROS-induced apoptosis. Therefore, NHLRC2 may be a possible drug target to sensitize cancer cells to ROS-dependent apoptosis induced by cancer chemotherapy.

In conclusion, the Trx-like domain protein NHLRC2 is a novel caspase-8 substrate that is involved in the regulation of ROS-induced apoptosis, and ROS-induced cleavage of NHLRC2 by caspase-8 leads to apoptotic cell death. Taken together, these results indicate the important role of NHLRC2 in ROS-induced apoptosis.

## Materials and methods

### Antibodies and constructs

Anti-FLAG (M2) (F1804) and anti-β-actin (A2066) antibodies were purchased from Sigma-Aldrich (St Louis, MO, USA). Peroxidase-conjugated anti-DYKDDDDK tag (015-22391) and peroxidase-conjugated anti-c-myc tag (014-21901) monoclonal antibodies were purchased from Wako Pure Chemical Industries (Osaka, Japan). Anti-NHLRC2 (ab88725) antibody was purchased from AbCam (Eugene, OR, USA). Anti-caspase-2 (11B4) antibody (MAB3507) was purchased from Millipore (Billerica, MA, USA). Anti-caspase-1 (3866), anti-caspase-3 (9665), anti-caspase-4 (4450), anti-caspase-6 (9762), anti-caspase-7 (12827), anti-caspase-8 (9746), anti-caspase-9 (9508), and anti-HA-tag (C29F4) (3724) antibodies were purchased from Cell Signaling Technologies (Danvers, MA, USA).

Human NHLRC2 complementary DNA was amplified from reverse-transcription products from HCT116 cells using the primers shown in Supplementary Table S1. The cDNAs encoding full-length or truncated forms of NHLRC2 tagged with HA at the N or C terminus were subcloned into a pCI-neo vector at XhoI and NotI sites using the primers shown in Supplementary Table S1. Several Asp residues in the NHLRC2 cDNA were mutated into asparagine using a QuickChange Site-Directed Mutagenesis kit (Takara Bio, Shiga, Japan). Sequences for forward primers used in the mutagenesis are shown in Supplementary Table S1. All expression vectors were verified by DNA sequencing.

The cDNAs for human caspases were amplified from reverse transcription products from HEK293 cells using the primers shown in Supplementary Table S1. Myc and FLAG tags were added at the N and C termini, respectively, of each caspase cDNA by PCR, and then myc-caspase-FLAG cDNAs were subcloned into a pcDNA3 vector at KpnI and NotI sites. The catalytic cysteine residues in caspase cDNAs were mutated into serine using a QuickChange Site-Directed Mutagenesis kit (Takara Bio). Sequences for the forward primers used in the mutagenesis are shown in Supplementary Table S1. All expression vectors were verified by DNA sequencing.

### Cells, treatment, and siRNA transfection

The HCT116 human colon cancer cell line and the HEK293 human embryonic kidney cell line (both from ATCC, Maryland, MD, USA) were cultured at 37 °C with 5% CO_2_ in Dulbecco’s Modified Eagle Medium (Wako Pure Chemical Industries), supplemented with 10% fetal calf serum and penicillin/streptomycin. For tBHP (458139, Sigma-Aldrich) or etoposide (E1383, Sigma-Aldrich) treatment, cells were seeded in 6- or 12-well plates at a density of 5 × 10^5^ and 2.5 × 10^5^ cells per well, respectively. After 24 h, the culture medium was switched to a medium containing chemical and the cells were incubated for a further 24 h. For scavenging ROS or inhibiting caspase activity, cells were preincubated for 1 h with NAC (A7250, Sigma-Aldrich) and z-VAD(OMe)-fmk (z-VAD-fmk, ab120487, AbCam), respectively.

The siRNAs against caspases were purchased from Invitrogen (Asheville, NC, USA). Sequences for each siRNA are shown in Supplementary Table S2. Cells were transfected with siRNA using Lipofectamine RNAiMAX (Invitrogen) according to the manufacturer’s reverse-transfection protocol. Briefly, cells were seeded with siRNA (10 pmol)–Lipofectamine RNAiMAX (5 µL) complexes in six-well plates at a density of 5 × 10^5^ cells per well. After 24 h, the culture medium was switched to a medium containing 300 µM tBHP, and the cells were incubated for a further 24 h.

### Cell lysates, immunoblotting, co-immunoprecipitation, and qRT-PCR

Cells were lysed and subjected to immunoblotting as described previously^46,47^. For co-immunoprecipitation, cells were lysed in Co-IP buffer (50 mM Tris-HCl, pH 7.5; 150 mM NaCl; 5 mM MgCl_2_; 10% glycerol and 1% NP-40) supplemented with cOmplete EDTA-Free Protease Inhibitor (Sigma-Aldrich) by incubation for 30 min at 4 °C. Cell pellets were removed by centrifugation and the supernatants were precleaned by incubation with rat or mouse IgG and Protein G Sepharose (GE Healthcare Life Sciences, Pittsburgh, PA, USA) for 30 min at 4 °C under gentle rotation. After centrifugation, the supernatants were co-immunoprecipitated by incubation with anti-HA affinity matrix (11815016001, Sigma-Aldrich), anti-DYKDDDDK tag antibody beads (018-22783, Wako Pure Chemical Industries), or anti-c-myc (9E10) antibody (sc-40, Santa Cruz Biotechnology, Santa Cruz, CA, USA) conjugated with Protein G Sepharose at 4 °C under gentle rotation overnight. The beads were washed with Co-IP buffer three times and then boiled in Laemmli sample buffer. The eluates were subjected to immunoblotting.

qRT-PCR was performed using the primers shown in Supplementary Table S1 as described previously^48^.

### Immunofluorescence microscopy

HEK293 cells seeded on to 12 mm diameter glass coverslips placed in 24-well plates were transfected with NHLRC2-HA expression vector alone or with myc-caspase-FLAG CS mutant vectors using Lipofectamine 3000 (Invitrogen). After 24 h, the transfected cells were fixed with 4% paraformaldehyde in phosphate-buffered saline (PBS) for 15 min, washed with PBS three times, and then permeabilized/blocked with 0.3% Triton X-100/5% non-fat dry milk in PBS for 30 min. For immunostaining, the cells were first incubated with anti-HA (C29F4) and anti-FLAG (M2) antibodies for 1 h at room temperature. Both antibodies were diluted in 0.3% Triton X-100/5% non-fat dry milk in PBS at a dilution of 1:1,000. Following incubation, cells were washed with PBS three times and subsequently incubated with Alexa Fluor 555 conjugated anti-mouse IgG and Alexa Fluor 488 conjugated anti-rabbit IgG antibodies (both from Invitrogen) at a dilution of 1:1,000 for 1 h at room temperature. Cells were washed with PBS three times, mounted in Fluorescence Mounting Medium (Dako, Santa Clara, CA, USA), and examined using a TCS SP5 laser-scanning confocal microscope (Leica Microsystems, Wetzlar, Germany).

### In vitro cleavage assay of NHLRC2 protein by caspases

293FT cells (Invitrogen) were transfected with expression vectors for wild-type or mutant NHLRC2-HA using polyethyleneimine “MAX” transfection reagent (Polysciences, Warrington, PA, USA) and then lysed in Cell Lysis Buffer (BioVision, Milpitas, CA, USA). Aliquots of lysates were incubated with 1 unit of recombinant active caspases (BioVision) in 1 × Reaction Buffer (BioVision) at 37 °C for 1 h. Reactions were terminated by adding Laemmli sample buffer and boiling at 95 °C for 5 min. Cleavage was evaluated by immunoblotting using an anti-NHLRC2 antibody.

### Production and transduction of retroviruses and lentiviruses

The oligonucleotides encoding shRNA against human NHLRC2, shown in Supplementary Table S3, were annealed and then inserted into the pSIREN-RetroQ-ZsGreen vector (Takara Bio) as described previously^49^. Negative control shRNA oligonucleotide was purchased from Takara Bio. GP2-293 retroviral packaging cells (Takara Bio) were transfected with both retroviral and pAmpho envelope vectors using a CalPhos Transfection kit (Takara Bio).

Oligonucleotides encoding sgRNA against human NHLRC2, shown in Supplementary Table S3, were annealed and then inserted into the pL-CRISPR.EFS.GFP vector (Addgene, Cambridge, MA, USA)^50^. Lentivirus was produced by transient transfection of 293FT cells using polyethyleneimine “MAX” transfection reagent. Viral constructs were cotransfected with pMDL, pRev and pVSVG vectors (kind gifts from Dr. Kinichi Nakashima, Kyushu University, Japan).

Culture supernatants containing retrovirus or lentivirus were collected 48 h after transfection. HCT116 cells were transduced with virus-containing medium supplemented with 8 µg/mL polybrene by centrifugation at 1,000 × *g* for 2 h at 32 °C and then further cultured for 8 h. The virus-containing medium was removed and replaced with fresh medium. After 3 days, ZsGreen or EGFP-positive cells were sorted using a FACSAriaII (BD Biosciences, San Jose, CA, USA) and reseeded in tissue culture plates for subsequent experiments.

### Caspase-8 activity measurement and cell proliferation assay

Cells were seeded in 96-well plates at a density of 5 × 10^3^ cells per well in 100 μL culture medium. After 24 h, the culture medium was switched to medium with or without tBHP. Caspase-8 activity and cellular proliferation were analyzed using Caspase-Glo8 assay (Promega, Madison, WI, USA) and Cell Counting Kit-8 (Dojindo, Kumamoto, Japan), respectively, according to the manufacturer’s protocols.

### Flow cytometry analysis of apoptotic cells

Cells were seeded in 24-well plates at a density of 2.5 × 10^4^ cells per well in 500 μL culture medium. After 24 h, the culture medium was switched to medium with or without tBHP or etoposide, and apoptotic cells were assessed using an APC Annexin V Apoptosis Detection kit (BD Biosciences). Flow cytometry data were acquired using a FACSAriaII and analyzed with FlowJo software (Tomy Digital Biology, Tokyo Japan) as described previously^48,51^.

### Statistical analysis

Data are expressed as the mean ± SD. Statistical analyses were performed using an unpaired two-tailed Student’s *t*-test. Differences at *P* < 0.05 were considered to be statistically significant.

## Electronic supplementary material

Supplemental Figure s1

Supplemental Figure s2

Supplemental Table s1

Supplemental Table s2

Supplemental Table s3
