# Supplementary figures and images for: ROS-induced cleavage of NHLRC2 by caspase-8 leads to apoptotic cell death in the HCT116 human colon cancer cell line
Source: Cell Death Dis. 2017 Dec 14;8(12):3218. doi: 10.1038/s41419-017-0006-7 (PMC5870588; doi:10.1038/s41419-017-0006-7)

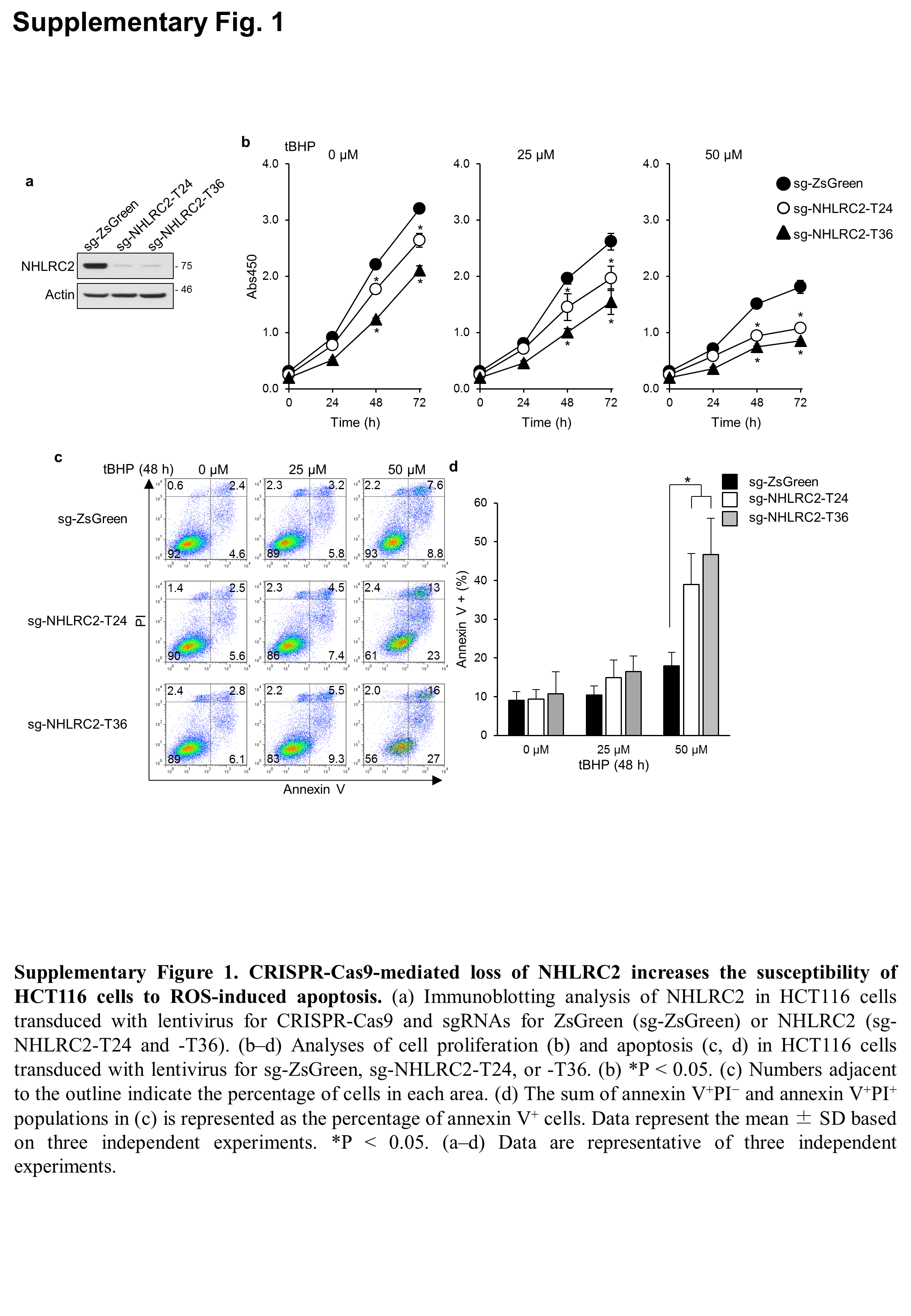

Supplement: Supplementary file 1 — Supplemental Figure s1 [file 41419_2017_6_MOESM1_ESM.tif]

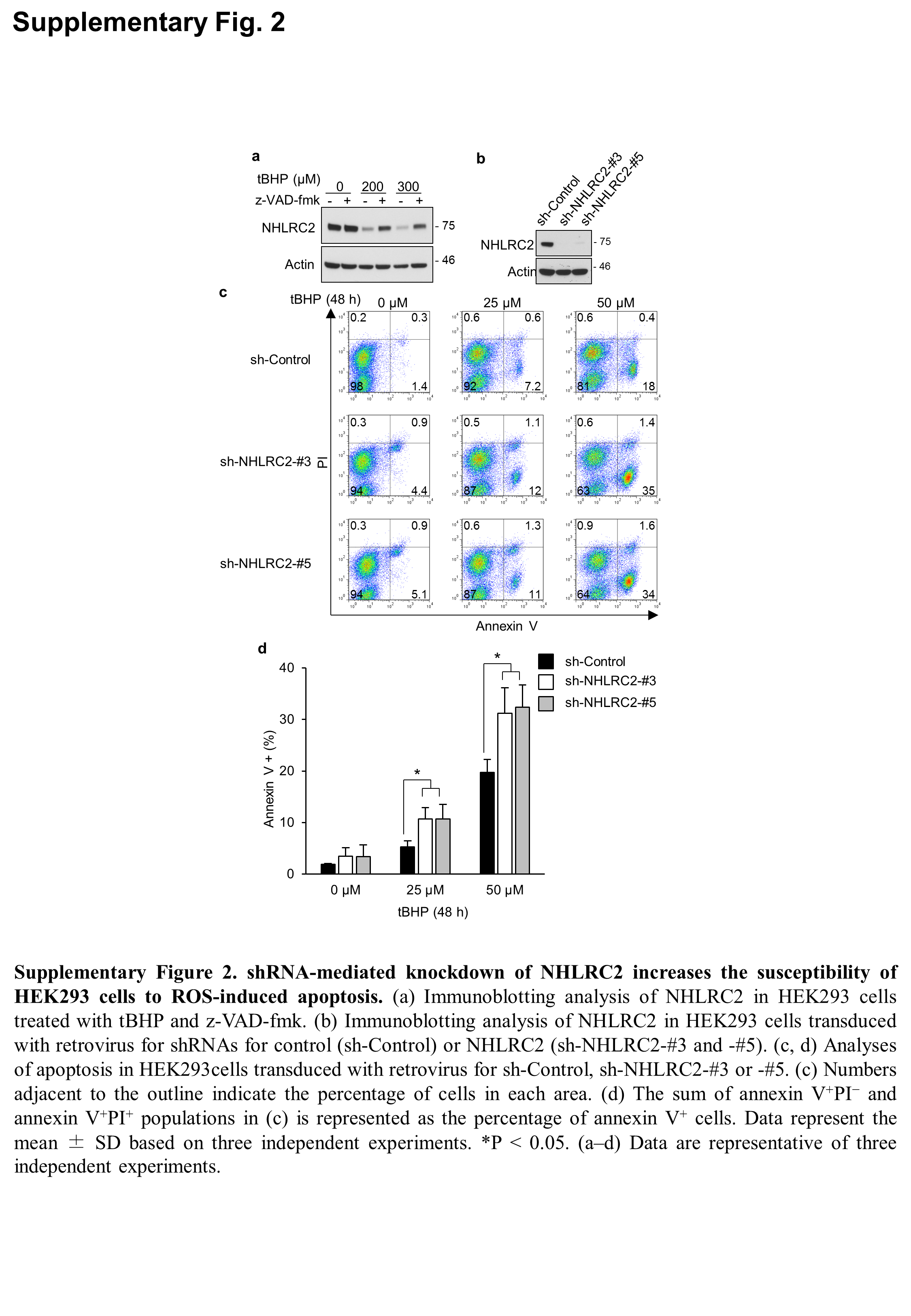

Supplement: Supplementary file 2 — Supplemental Figure s2 [file 41419_2017_6_MOESM2_ESM.tif]
