# Supplementary material for: ROS-induced cleavage of NHLRC2 by caspase-8 leads to apoptotic cell death in the HCT116 human colon cancer cell line
Source: Cell Death Dis. 2017 Dec 14;8(12):3218. doi: 10.1038/s41419-017-0006-7 (PMC5870588; doi:10.1038/s41419-017-0006-7)
Supplement: Supplementary file 3 — Supplemental Table s1 [file 41419_2017_6_MOESM3_ESM.docx]

Supplemental Table s1

Primer sequences used in this study.

| Primer | Sequence (5’ to 3’) |
| --- | --- |
| hNHLRC2-f2 | aacccattagttccccttgg |
| hNHLRC2-r2 | cagcaggagtgcccatatct |
| hACTB-f | caccaactgggacgacat |
| hACTB-r | acagcctggatagcaacg |
| hNHLRC2-N1 | atggcggcgcccggaggccggg |
| hNHLRC2-C1 | aaatacatacctgagctctactg |
| hNHLRC2-N2 | tttctcgaggccgccaccatggcggcgcccggaggccgg |
| hNHLRC2-C2 | aaagcggccgcctaaaatacatacctgagctctac |
| HA-hNHLRC2-N | tttctcgaggccgccaccatgtacccatacgatgttccagattacgctgcggcgcccggaggccggggccgcagcctctcc |
| hNHLRC2-HA-C | aaagcggccgcctaagcgtaatctggaacatcgtatgggtaaaatacatacctgagctctactggagctatgca |
| Trx-C | aaagcggccgcctatccctctccaatcaaagaaaa |
| HA-NHL-N | tttctcgaggccgccaccatgtacccatacgatgttccagattacgctggaataaaactctataaagattctttgccacct |
| NHLRC2-D27Nf | gagtacgccctgctcaacgccgttacccag |
| NHLRC2-D111Nf | acatactctgataaaaatggtcttcttatt |
| NHLRC2-D295Nf | atcatatatgtggcaaacactgaaaaccac |
| NHLRC2-D430Nf | tgcttgtttgtagcaaatagtgagagcagt |
| NHLRC2-D443Nf | accgtttcactgaaaaatggagcagtgaag |
| NHLRC2-D466Nf | gcttttggtgatgttaatggagtaggaatc |
| NHLRC2-D493Nf | ttactttatgttgcaaactcctacaatcac |
| NHLRC2-D550Nf | ttattatatgtagcaaacaccaataatcat |
| NHLRC2-D580Nf | gaaaatgctgtggtaaatggcccgttccta |
| NHLRC2-D692Nf | tattactgtagtgcaaacagcagtgcttgt |
| CASP1-N2 | atggccgacaaggtcctgaagg |
| CASP1-C2 | atgtcctgggaagaggtagaaacatc |
| CASP2-N2 | atggcggcgccgagcgcgggg |
| CASP2-C2 | tgtgggagggtgtcctgggaacag |
| CASP3-N2 | atggagaacactgaaaactcagtgg |
| CASP3-C2 | gtgataaaaatagagttcttttgtgagc |
| CASP4-N2 | atggcagaaggcaaccacagaaaaaag |
| CASP4-C2 | attgccaggaaagaggtagaaatatcttg |
| CASP6-N2 | atgagctcggcctcggggctc |
| CASP6-C2 | attagattttggaaagaaatgcagctttttag |
| CASP7-N2 | atggcagatgagcagggctgtattg |
| CASP7-C2 | ttgactgaagtagagttccttggtg |
| CASP8-N2 | atggacttcagcagaaatctttatgatat |
| CASP8-C3 | atcagaagggaagacaagtttttttcttag |
| CASP9-N2 | atggacgaagcggatcggcggc |
| CASP9-C2 | tgatgttttaaagaaaagttttttccgg |
| CASP10-N2 | atgaaatctcaaggtcaacattggtattc |
| CASP10-C2 | taatgaaagtgcatccaggggcacagg |
| CASP1_CSf | atcatccaggccagccgtggtgacagc |
| CASP2_CSf | cttcatccaggccagccgtggagatgagac |
| CASP3_CSf | attattcaggccagccgtggtacagaa |
| CASP4_CSf | attgtccaggccagcagaggtgcaaac |
| CASP6_CSf | atcattcaggcaagtcggggaaaccag |
| CASP7_CSf | ttcattcaggctagccgagggaccgag |
| CASP8_CSf | tttattcaggctagtcagggggataac |
| CASP9_CSf | ttcatccaggccagtggtggggagcag |
| CASP10_CSf | ttcatccaggccagccaaggtgaagag |
