# Supplementary material for: ROS-induced cleavage of NHLRC2 by caspase-8 leads to apoptotic cell death in the HCT116 human colon cancer cell line
Source: Cell Death Dis. 2017 Dec 14;8(12):3218. doi: 10.1038/s41419-017-0006-7 (PMC5870588; doi:10.1038/s41419-017-0006-7)
Supplement: Supplementary file 4 — Supplemental Table s2 [file 41419_2017_6_MOESM4_ESM.docx]

| CASPs | Description | Sequence (5’ to 3’) |
| --- | --- | --- |
| CASP-2 | CASP2HSS141454 | UGCACGUGGCCGACAUGCUGGUUAA |
| CASP-3 | CASP3HSS101371 | GGAAUAUCCCUGGACAACAGUUAUA |
| CASP-4 | CASP4HSS141458 | GGGAAUCUGCGGAACUGUGCAUGAU |
| CASP-6 | CASP6HSS101378 | GCCAGUCAUUCCUUUGGAUGUAGUA |
| CASP-7 | CASP7HSS101382 | UCCACGGUUCCAGGCUAUUACUCGU |
| CASP-8 | CASP8HSS141461 | CCCAUCAAGGAUGCCUUGAUGUUAU |
| CASP-9 | CASP9HSS141465 | UGCGAACUAACAGGCAAGCAGCAAA |

Supplemental Table s2

siRNA sequences used in this study
