# Supplementary material for: ROS-induced cleavage of NHLRC2 by caspase-8 leads to apoptotic cell death in the HCT116 human colon cancer cell line
Source: Cell Death Dis. 2017 Dec 14;8(12):3218. doi: 10.1038/s41419-017-0006-7 (PMC5870588; doi:10.1038/s41419-017-0006-7)
Supplement: Supplementary file 5 — Supplemental Table s3 [file 41419_2017_6_MOESM5_ESM.docx]

Supplemental Table s3

Oligonucleotide sequences for shRNA and sgRNA used in this study

|  | Sequence (5’ to 3’) |
| --- | --- |
| shRNA |  |
| hNHLRC2-3f | gatccggacaaattcaatatagcatttcaagagaatgctatattgaatttgtccttttttg |
| hNHLRC2-3r | aattcaaaaaaggacaaattcaatatagcattctcttgaaatgctatattgaatttgtccg |
| hNHLRC2-5f | gatccgcctacaatcacaagattaaagtcaagagctttaatcttgtgattgtaggttttttg |
| hNHLRC2-5r | aattcaaaaaacctacaatcacaagattaaagctcttgactttaatcttgtgattgtaggcg |
| sgRNA |  |
| hNHLRC2-T24f | caccgcgtccaccttctgcagatac |
| hNHLRC2-T24r | aaacgtatctgcagaaggtggacgc |
| hNHLRC2-T36f | caccgtcagtacccgagtttccgga |
| hNHLRC2-T36r | aaactccggaaactcgggtactgac |
| ZsGreen-f | caccgaccatgaagtaccgcatgg |
| ZsGreen-r | aaacccatgcggtacttcatggtc |
